# Associations between Adverse Childhood Experiences and Emergency Department Utilization in an Adult Medicaid Population

**DOI:** 10.3390/ijerph191610149

**Published:** 2022-08-16

**Authors:** Kristin Lyon-Scott, Hannah Cohen-Cline

**Affiliations:** 1 OCHIN, Inc., Portland, OR 97228, USA; 2 Providence Center for Outcomes Research and Education, Portland, OR 97213, USA

**Keywords:** adverse childhood experiences, emergency department utilization, social support, Medicaid

## Abstract

Adverse childhood experiences (ACEs) are widely prevalent but unevenly distributed in the United States, with disadvantaged groups, especially those with low socioeconomic status, being more likely to experience them. ACEs have been linked to poor health outcomes in adulthood. In this study, we examined the association between ACEs and emergency department (ED) utilization using a cross-sectional life-course survey of low-income adults matched to Medicaid enrollment and claims data. Surveys were obtained from 2348 Medicaid-enrolled adults in the Portland, OR metropolitan area; 1133 were used in this analysis. We used a two-part regression model to estimate the association between ACE score and both ever using the ED and frequency of ED use in the year after survey completion. We also evaluated a set of potentially protective factors to see if they impacted the relationship between ED use and ACE score. We found that participants with a higher ACE score were more likely to obtain any emergency services care (odds ratio (OR) = 1.11, *p* = 0.011), but ACE score did not predict how frequently they would utilize those services. Close social relationships were found to be protective against high ED utilization for those with high ACE scores. Upstream prevention efforts that identify places to intervene in childhood and incorporate trauma-informed strategies into ED care in adulthood have the potential to decrease ED use.

## 1. Introduction

Adverse childhood experiences (ACEs) are widespread in the United States, with approximately two-thirds of adults reporting having experienced at least one ACE [1]. The prevalence of ACEs is not evenly distributed among the population, however; there is an increased prevalence among more disadvantaged populations [2], including individuals with lower socioeconomic status (SES) [3,4,5]. This uneven distribution of ACEs is of critical importance to public health, in part because of extensive previous research linking ACEs to a plethora of poor health outcomes in adulthood, including an increased likelihood of heart disease, cancer, mental health concerns, including abuse of alcohol and other substances, serious injury, and premature mortality [6,7,8,9,10,11,12]. In addition, individuals with a low SES often have fewer resources available to cope with trauma or its resulting health concerns, potentially worsening the long-term effects of ACEs in this population [13].

Emergency department (ED) utilization is also substantially more prevalent among low-SES individuals [14,15]. Previous research has suggested different potential explanations for this. Lower SES may lead to poorer health, which in turn leads to increased use of acute health care services, such as the ED [16]. Individuals with lower SES may also encounter more logistical barriers in navigating the fragmented health care system, as well as financial barriers such as a lack of health insurance, which then forces them to use the ED for care that could otherwise have been provided in an outpatient setting [17,18,19,20]. However, studies in countries with universal health coverage, as well as those that adjust for insurance status, have still shown an association between SES and ED use, suggesting the presence of factors other than a lack of insurance [21,22,23].

High ED utilization is problematic for both patients and health systems. When used in place of ambulatory care, ED use can lead to a lack of continuity of care, delays in seeking needed care, and limited ability to manage chronic conditions, all of which can further widen health disparities already experienced by low-income populations [24,25]. For health systems, ED use is more expensive and consumes more resources than primary care, leading to higher expenditures and growing costs of care [26,27]. It is therefore critical to understand what drives ED use, especially among the low-SES population. One possible explanation is the increased prevalence of ACEs in the low SES-population, which may contribute to poorer health and increased barriers to care.

Some previous studies have explored the association between ACEs and ED utilization, often either among specific age groups or individuals with specific chronic conditions, with mixed results. A study by Diaz et al. showed an association between higher ACE scores and more frequent and lower acuity ED visits among adults, even after adjusting for sociodemographic factors [28]. A study by Carbone et al. showed that ACEs were associated with increased emergency department visits for self-injury and suicide attempts among children and adolescents [29]. In contrast, Bhattarai et al. found that ACEs did not predict future ED use among children and adolescents with pre-existing mental health conditions [30]. Additionally, Murray et al. found that a history of ACEs among parents and caregivers was not associated with low acuity ED use for their children [31].

In this study, we examined the association between ACEs and ED utilization in a low-income population in the Portland, OR metropolitan area. We hypothesized that higher ACE scores would lead to both a greater likelihood of having any ED visit, as well as an increased number of ED visits. We further explored a set of potentially protective factors, hypothesizing that some characteristics or experiences might mitigate the association between ACEs and ED utilization.

## 2. Materials and Methods

### 2.1. Survey Development and Sample Selection

We developed and fielded a cross-sectional life-course survey among adult Medicaid enrollees in the Portland, OR metropolitan area. The survey domains were developed from 72 previous detailed qualitative interviews we had conducted among Medicaid members in order to identify and understand frequent and/or important formative experiences across the life course. These domains ultimately included relationships and social support, educational challenges, family housing and employment stability, neighborhood environment, discrimination, abuse, and household dysfunction. Questions were asked across three age ranges: early childhood (0–5 years of age), later childhood (6–12 years of age), and adolescence (13–18 years of age). More details on the creation of the survey can be found in previous publications [32,33].

We fielded the survey in 2015 and 2016. Medicaid members were eligible to receive a survey if they were 18–65 years old, had a valid mailing address, and were enrolled in Medicaid for at least 6 months in the year prior to survey fielding. We oversampled Black/African Americans, enrollees with high medical complexity, and enrollees with frequent use of acute health care. High medical complexity was defined as the top 10th percentile of the Chronic Illness and Disability Payment System (CDPS) scores, a claims-based algorithm that scores members on demographics, chronic medical conditions, and prescription patterns [34]. Frequent acute health care utilization was defined as 3 or more emergency room visits, 2 or more inpatient stays, or 2 emergency department visits and 1 inpatient stay in the year prior to survey fielding.

Surveys were sent to a total of 9176 individuals. To encourage higher response rate, potential respondents received a USD 5 incentive, as well as follow-up mailings and phone calls, and a random subset of 22% of the sample received door-to-door outreach. We received a total of 2348 responses. To be included in the current analysis, survey respondents needed to have at least 6 consecutive months of Medicaid enrollment following survey completion, and complete survey data on all key variables. This resulted in a final analytic sample of 1133 survey respondents. 

### 2.2. Measures

Our main independent variable for this analysis is ACE score, based on the original conceptualization of ACEs developed by Felitti et al. in his seminal work in the Kaiser Permanente population [8]. This score ranges from 0 to 10, with higher scores indicating the presence of more ACEs. It covers ten experiences across three domains: abuse (physical, emotional, sexual); neglect (physical, emotional); and household dysfunction (mentally ill household member, household member with substance abuse concerns, household member in jail/prison, witnessing violence between caregivers, and divorce). ACE score was treated as a continuous variable in the analysis.

Our key health care outcomes were constructed from Medicaid claims data. ED utilization was included both as a binary variable (ever/never in the year after survey fielding) and as the average number of visits per member per year. ED visits resulting in inpatient stays were excluded from analyses, as these are generally classified as hospitalizations. 

We included several sociodemographic covariates in our models. Age at time of survey completion (years), sex (female, male), and race (White, Black/African American, Asian, Multi-race, or “Other”) were obtained from the survey; if data were missing on the survey, they were obtained from the Medicaid enrollment files. Income was represented by the percent Federal Poverty Level (FPL) and was calculated from self-reported income and household size on the survey. FPL was categorized as less than or equal to 100%, 101–138%, 139–150%, 151–200%, and 201% or more. Educational attainment was categorized as less than high school education, high school diploma or GED, some college or vocational training, or a 4-year college degree or more.

Finally, we included a set of potentially protective factors that we hypothesized could mitigate any observed association between ACE scores and ED utilization. All factors were obtained from the survey and included whether the participant has a personal doctor; if the participant trusts their personal doctor; involvement in formal social groups; and informal social support.

### 2.3. Statistical Analysis

Because ED utilization is zero-inflated and heavily right-skewed, we used a two-part model to assess its association with ACE score [35]. The two-part model simultaneously models ED utilization as a binary outcome (ever/never) using logistic regression and, for those who had an ED visit, as a continuous outcome using a generalized linear model (GLM) with a gamma distribution. We further assessed effect modification by sex and race, hypothesizing that ACE score may impact ED utilization differently for different demographic groups.

We next used logistic regression to compare our set of potentially protective factors among respondents with high ACE scores and low ED utilization, versus respondents with high ACE scores and high ED utilization, to assess if the odds of these factors were different between the two groups. A high ACE score was defined as a score of 4 or more, a commonly used threshold for high ACEs [36,37,38]. High ED utilization was defined as 3 or more visits in the 12-month study period.

All analyses were adjusted for sex, age, race, education, and FPL, and weighted to account for the survey sampling methods. All statistical analysis was performed using STATA (V 14.0, StataCorp, College Station, TX, USA), with two-sided statistical significance tests set at *p* < 0.05. All study procedures were reviewed and approved by the Providence Institutional Review Board.

## 3. Results

Table 1 gives the characteristics of the study sample, stratified by ED utilization. Of the 1133 survey respondents included in the analysis, 205 (18.1%) had three or more ED visits in the 12 months following survey completion. Respondents with high ED utilization had higher mean ACE scores (4.4 vs. 3.3 ACEs), were more likely to identify as female and “Other” race, and had lower educational attainment and income, compared to respondents with lower ED utilization. There were no substantial differences in age between the two groups.

Table 2 presents the results from the two-part model. The adjusted odds ratio (OR) from the logistic regression part of the model was 1.11 (95% confidence interval (CI): 1.02, 1.21; *p* = 0.011), suggesting that each additional point on the ACE score was associated with 11% increased odds of having at least one ED visit in the year after completing the survey. In the GLM, the adjusted coefficient for the ACE score was 0.026, but was not statistically significant (95% CI: −0.02, 0.07; *p* = 0.228). This suggests that, for individuals with at least one ED visit, ACE score has no impact on the total number of ED visits per year.

We tested for effect modification by sex and race in both the full two-part model (data not shown), as well as in the logistic regression model by itself (Table 3). In both models, the interaction terms were not significant, suggesting that the associations between ACE score and ED utilization do not differ across these two demographic characteristics.

Table 4 presents the results from the analysis of potentially protective factors. Among individuals with an ACE score of 4 or greater (N = 1146), we did not observe a statistically significant association between ED utilization and having and/or trusting a personal doctor or participating in formal social groups. In contrast, several of the measures of informal social support were associated with decreased odds of ED utilization, including: having someone to love you and make you feel wanted (OR: 1.87; 95% CI: 1.21, 2.88; *p* = 0.004); having someone available to ask for advice during a crisis (OR: 1.62; 95% CI: 1.19, 2.22; *p* = 0.002); and having someone to get together with for relaxation (OR: 2.07; 95% CI: 1.35, 3.15; *p* = 0.001). 

## 4. Discussion

In the current study, we found a strong association between ACE scores and having at least one ED visit in an adult Medicaid population, with each additional point on the ACE score associated with 11% increased odds of having an ED visit. In contrast, we did not observe any association between ACE score and ED visit frequency, nor did we find evidence that the observed association was modified by race or sex. 

We present two potential explanations as to why ACE score may be associated with visiting the ED at all during a 12-month period, but not with the total number of visits. First, it may indicate that individuals are using the ED for truly emergent one-off health needs such as injuries, and then transitioning to outpatient care for follow-up. This is supported by previous research that linked higher ACEs to increased risk for injuries, including both accidental injuries and victimization [10,39]. 

Alternatively, individuals with higher ACE scores may be more likely to use the ED in replacement of outpatient care due to logistical or preference barriers to primary care, but not due to increased severity of illness. There has been substantial previous research assessing the major reasons for which individuals seek care through the ED, especially for non-emergent health needs. A 2017 systematic literature review identified six distinct reasons individuals used the ED; of these themes, the top four reasons—limited access to primary care, convenience, patient anxiety, and patient-specific barriers like cost or lack of transportation—are not associated with greater health needs or severity of symptoms [40]. Interestingly, research specifically among lower-SES individuals has also found that those who use the ED or hospital care often do so because they perceive it to be higher quality than outpatient care [41,42].

We had hypothesized that a higher ACE score would be associated with a greater number of ED visits, given previous research that has shown an association between high ACEs and poor health in adulthood [6,7,8,9,10,11,12]. Under this hypothesis, we would expect to see an association between higher ACE score and a greater number of ED visits per year, as patients would be continually seeking acute care support. However, we did not observe the hypothesized association between ACE score and ED visit frequency. Further, when we explored the association between ACEs and medical complexity as measured by the CDPS in our own data, we found similar levels of medical complexity between individuals with high ACEs (ACE score ≥ 4) and without high ACEs (data not shown). Although there is little previous research on the association between ACE score specifically and frequency of ED use, studies that have explored associations between social and economic factors and frequent ED use often find that these factors drive ED utilization when in conjunction with an increased health burden [43,44,45,46]. Our lack of association between ACE score and ED visit frequency may therefore be due to the similar levels of medical complexity between the groups, and future research should explore interactions between ACE score and health in driving associations with acute health care utilization. 

Understanding why individuals with higher ACE scores are more likely to use the ED is key to devising policy and practice interventions, and future research should explore this, whether through analyzing the diagnosis codes associated with ED visits or conducting patient surveys. For instance, if this association is primarily due to an increased risk of injuries as hypothesized above, focusing upstream prevention efforts on preventing injuries could be a critical step in reducing ED use for this population. Further research to understand the type of injury would be beneficial to guide those prevention efforts, as, for example, interventions to prevent violence and victimization would certainly be different than interventions to prevent car crashes.

Intervention strategies could also focus on identifying points of influence during an individual’s childhood. Screening for ACEs in the pediatric population to identify points of leverage may be impactful in preventing or mitigating risky health behaviors and high-cost utilization patterns in adulthood. For example, previous research has identified social workers as an important resource for this population with the potential to deliver health literacy interventions in multiple healthcare settings [47].

In addition, hospital systems and staff should incorporate both assessing a history of ACEs, and understanding reasons for using the ED, into ED discharge planning to address individual needs. The use of trauma-informed care in this setting is critical to avoiding retraumatizing individuals in the ED and ensuring that they are connected to appropriate follow-up care [48,49,50]. This may include providing individuals with connections outside of the clinical setting, as community-based interventions have been shown to support building strengths-based behaviors that can encourage better health [51]. 

Future research should explore if and how this observed association may differ across different sociodemographic groups and geographic locations. Although we did not observe any differences in the association by sex or race, it is possible that the mechanisms underlying this association may be different for different sexes and racial/ethnic groups. There may also be differences by characteristic we did not explore. For example, individuals with higher levels of education may be more able to navigate complicated health care systems than those with a lower level of education; or those living in urban areas may have more access to primary or preventive care, compared to their rural-based counterparts. Understanding these differences and how they may impact both the observed association between ACEs and ED use, as well as the underlying mechanisms, is key to designing appropriate intervention strategies.

In this study, we additionally found that having emotionally close personal relationships and social support is protective against high ED utilization among those with high ACEs, while there was no impact of participation in formal social activities. These findings are aligned with the robust body of literature showing strong associations between social support and improved health [52]. This is particularly important considering the current COVID-19 pandemic, which has increased experiences of social isolation in the general population. As evidence suggests, these relationships are important for improved health and health care, and thus upstream efforts to help individuals maintain and rebuild them during a crisis such as the global pandemic could have a substantial impact on downstream health outcomes.

These findings should be interpreted in the context of the study’s limitations. This study was completed among an adult Medicaid-enrolled population in a large metropolitan area, which may limit generalizability to more affluent populations in other geographical areas. However, given that the goal of this study was to understand the association between ACE score and ED utilization specifically within a low-income population, the lack of generalizability to wealthier populations is less of a concern. The study population being urban does prevent us from understanding if this association may look different in rural areas, where individuals often have less access to health care and to other resources to address social needs. Additionally, the study population is predominantly white, and while this reflects the general population of the Portland, OR metropolitan area, it does limit generalizability to more demographically diverse populations. In particular, experiences within and perceptions of the health care system have been shown to differ based on race, with Black, Indigenous, and People of Color (BIPOC) individuals experiencing increased discrimination and barriers to care, which impact their health care utilization patterns [53,54,55]. Finally, our survey data may be subject to both response and recall bias. Individuals may have difficulty recalling traumatic experiences or may perceive past trauma through the lens of current trauma [56].

## 5. Conclusions

The results of this study suggest a connection between experiences of adversity in childhood and ED utilization among a low-income population, as well as provide evidence for the protective effects of informal social support. Building upstream prevention efforts that identify places to intervene in both childhood and adulthood, as well as incorporating trauma-informed strategies into ED care, could potentially decrease the use of the ED in this population, which may lead to improved connections to more appropriate health care and enhance health outcomes. Future research should continue to explore the connections between ACEs and ED utilization to understand, and interrupt, the mechanisms underlying this association.

## Figures and Tables

**Table 1:** Socioeconomic characteristics of the study population of Medicaid-enrolled adults in the Portland, OR metropolitan area, stratified by high and low ED utilization.

	High ED ^1^ N = 205		Low ED ^1^ N = 928	
	N	%	N	%
ACE Score ^2^	4.4	0.44	3.3	0.15
Age ^2^	43.2	2.22	44.5	0.69
Sex				
Female	149	77.65	563	62.39
Male	56	22.35	365	37.61
Race				
Asian	0	0.00	46	7.91
Black/African American	24	7.49	108	5.45
Multiracial	18	5.54	69	6.92
Other	14	9.41	35	4.79
White	149	77.57	670	74.93
Education				
Less than high school	36	25.38	136	11.41
High school diploma/GED	72	34.36	320	28.53
Some college or vocational training	75	33.95	354	42.14
4-year college degree or more	22	6.31	118	17.92
Federal Poverty Level				
Less than 100%	169	82.86	710	69.65
101–138%	18	5.42	98	14.64
139–150%	6	7.50	32	5.30
151–200%	8	3.10	41	4.16
201% or more	4	1.13	47	6.25

^1^ High ED utilization defined as having 3 or more ED visits in the 12-month period post survey completion. Low ED utilization defined as having less than 3 visits in the 12-month period. ^2^ Mean and standard deviation.

**Table 2:** Two-part model of associations between ACE score and ED utilization within 12 months of survey completion.

**Logistic Regression**	**Adjusted OR**	**95% CI**	***p*-Value**
ACE score	1.11	1.02, 1.21	0.011
Age	1.00	0.98, 1.02	0.819
Sex (male)	1.44	0.89, 2.34	0.139
Race			
Asian	0.50	0.15, 1.69	0.265
Black/African American	3.07	1.56, 6.03	0.001
Multiracial	1.29	0.60, 2.79	0.511
Other	1.75	0.65, 4.71	0.270
White	1.00	*ref*	*ref*
Education			
Less than high school	1.00	*ref*	*ref*
High school diploma/GED	0.62	0.28, 1.40	0.251
Some college or vocational training	0.52	0.23, 1.18	0.117
4-year college degree or more	0.36	0.14, 0.95	0.039
Federal Poverty Level			
Less than 100%	1.00	*ref*	*ref*
101–138%	0.80	0.38, 1.71	0.564
139–150%	1.03	0.34, 3.08	0.958
151–200%	3.03	0.98, 9.37	0.054
201% or more	2.00	0.52, 7.73	0.317
**GLM Regression**	**Adjusted β**	**95% CI**	***p*-value**
ACE score	0.026	0.07, 0.23	0.228
Age	0.009	0.00, 0.02	0.040
Sex (male)	0.072	−0.15, 0.29	0.519
Race			
Asian	−0.578	−1.07, −0.09	0.021
Black/African American	−0.269	−0.49, −0.05	0.018
Multiracial	−0.064	−0.43, 0.30	0.734
Other	0.188	−0.14, 0.52	0.260
White	1.00	*ref*	*ref*
Education			
Less than high school	1.00	*ref*	*ref*
High school diploma/GED	−0.013	−0.29, 0.26	0.926
Some college or vocational training	−0.099	−0.41, 0.22	0.538
4-year college degree or more	−0.211	−0.55, 0.13	0.219
Federal Poverty Level			
Less than 100%	1.00	*ref*	*ref*
101–138%	−0.140	−0.43, 0.15	0.350
139–150%	−0.339	−0.96, 0.28	0.285
151–200%	−0.303	−0.60, −0.01	0.042
201% or more	−0.660	−1.05, −0.27	0.001

**Table 3:** Associations between ACE score and having at least one ED visit within the 12 month study window, interaction by sex and race.

	Adjusted OR ^1^	95% CI	*p*-Value
ACE Score	1.04	0.92, 1.17	0.581
Sex (male)	0.91	0.43, 1.91	0.796
ACE × Sex interaction	1.13	0.97, 1.33	0.129
ACE Score	1.12	1.02, 1.23	0.018
Race			
Asian	0.73	0.13, 4.17	0.722
Black/African American	4.25	1.53, 11.789	0.005
Multiracial	0.85	0.16, 4.537	0.850
Other	1.76	0.41, 7.594	0.452
White	1.00	ref	ref
ACE × Race interaction			
Asian	0.58	0.20, 1.64	0.304
Black/African American	0.89	0.71, 1.10	0.274
Multiracial	1.08	0.79, 1.48	0.638
Other	0.98	0.73, 1.33	0.917
White (Ref)	1.00	ref	ref

^1^ Adjusted for age, education, Federal Poverty Level, and sex or race.

**Table 4:** Associations between hypothesized protective factors and having at least one ED visit in the 12 months after survey completion among survey respondents with high ACE scores (ACE ≥ 4).

	Adjusted OR ^1^	95% CI	*p*-Value
Social groups			
Involvement in educational or school group	0.49	0.11, 2.15	0.346
Involvement in church or religious group	1.00	0.43, 2.31	0.995
Involvement in civic or community group	1.05	0.38, 2.92	0.930
Involvement in recreational group or sports club	0.57	0.18, 1.77	0.329
Social support			
Had someone to love you or make you feel wanted	1.87	1.21, 2.88	0.004
Had someone available for good advice during crisis	1.62	1.19, 2.22	0.002
Had someone to relax with	2.07	1.35, 3.15	0.001
Had someone to confide in	1.28	0.79, 2.09	0.314
Had someone to help if confined to a bed	1.73	1.26, 2.37	0.001
Healthcare			
Had a personal doctor	1.21	0.20, 7.41	0.838
Had a personal doctor you could trust	1.31	0.54, 3.17	0.554

^1^ Adjusted for age, education, Federal Poverty Level, sex, and race.

## Data Availability

The survey data presented in this study are available on request from the corresponding author, with appropriate Ethics Committee or Institutional Review Board review and approval. Restrictions apply to the availability of the claims data. Data was obtained from Health Share of Oregon and are available with permission of Health Share of Oregon (https://www.healthshareoregon.org/).

## References

[1] Centers for Disease Control and Prevention, US Department of Health and Human Services Behavioral Risk Factor Surveillance System. https://www.cdc.gov/brfss/index.html.

[2] Merrick M.T., Ford D.C., Ports K.A., Guinn A.S. (2018). Prevalence of Adverse Childhood Experiences from the 2011–2014 Behavioral Risk Factor Surveillance System in 23 States. JAMA Pediatr..

[3] Mersky J.P., Janczewski C.E., Topitzes J. (2017). Rethinking the Measurement of Adversity: Moving toward Second-Generation Research on Adverse Childhood Experiences. Child Maltreat..

[4] O’Connor C., Finkbiner C., Watson L. (2012). Adverse Childhood Experiences in Wisconsin: Findings from the 2010 Behavioral Risk Factor Survey.

[5] Tilson E.C., Carolina N. (2018). Adverse Childhood Experiences (ACEs). N. C. Med. J..

[6] Brown M.J., Thacker L.R., Cohen S.A. (2013). Association between Adverse Childhood Experiences and Diagnosis of Cancer. PLoS ONE.

[7] Dong M., Anda R.F., Dube S.R., Giles W.H., Felitti V.J. (2003). The Relationship of Exposure to Childhood Sexual Abuse to Other Forms of Abuse, Neglect, and Household Dysfunction during Childhood. Child Abuse Negl..

[8] Felitti V.J., Anda R.F., Nordenberg D., Williamson D.F., Spitz A.M., Edwards V., Koss M.P., Marks J.S. (1998). Relationship of Childhood Abuse and Household Dysfunction to Many of the Leading Causes of Death in Adults: The Adverse Childhood Experiences (ACEs) Study. Am. J. Prev. Med..

[9] Gilbert L.K., Breiding M.J., Merrick M.T., Thompson W.W., Ford D.C., Dhingra S.S., Parks S.E. (2015). Childhood Adversity and Adult Chronic Disease: An Update from Ten States and the District of Columbia, 2010. Am. J. Prev. Med..

[10] Ports K.A., Ford D.C., Merrick M.T. (2016). Adverse Childhood Experiences and Sexual Victimization in Adulthood. Child Abuse Negl..

[11] Whitfield C., Anda R., Dube S., Felitti V. (2003). Violent Childhood Experiences and the Risk of Intimate Partner Violence in Adults: Assessment in a Large Health Maintenance Organization. J. Interpers. Violence.

[12] Anda R.F., Felitti V.J., Bremner J.D., Walker J.D., Whitfield C., Perry B.D., Dube S.R., Giles W.H. (2006). The Enduring Effects of Abuse and Related Adverse Experiences in Childhood: A Convergence of Evidence from Neurobiology and Epidemiology. Eur. Arch. Psychiatry Clin. Neurosci..

[13] Cronholm P.F., Forke C.M., Wade R., Bair-Merritt M.H., Davis M., Harkins-Schwarz M., Pachter L.M., Fein J.A. (2015). Adverse Childhood Experiences: Expanding the Concept of Adversity. Am. J. Prev. Med..

[14] Tang N., Stein J., Hsia R.Y., Maselli J.H., Gonzales R. (2010). Trends and Characteristics of US Emergency Department Visits, 1997–2007. JAMA.

[15] Chiu Y.M., Vanasse A., Courteau J., Chouinard M.C., Dubois M.F., Dubuc N., Elazhary N., Dufour I., Hudon C. (2020). Persistent Frequent Emergency Department Users with Chronic Conditions: A Population-Based Cohort Study. PLoS ONE.

[16] Winkleby M.A., Jatulis D.E., Frank E., Fortmann S.P. (1992). Socioeconomic Status and Health: How Education, Income, and Occupation Contribute to Risk Factors for Cardiovascular Disease. Am. J. Public Health.

[17] Brite J., Alper H.E., Friedman S., Takemoto E., Cone J. (2020). Association between Socioeconomic Status and Asthma-Related Emergency Department Visits among World Trade Center Rescue and Recovery Workers and Survivors. JAMA Netw. Open.

[18] Van Stone N.A., Belanger P., Moore K., Caudle J.M. (2014). Socioeconomic Composition of Low-Acuity Emergency Department Users in Ontario. Can. Fam. Physician.

[19] Newton M.F., Keirns C.C., Cunningham R., Hayward R.A., Stanley R. (2008). Uninsured Adults Presenting to US Emergency Departments: Assumptions vs. Data. JAMA.

[20] Schoen C., Doty M.M. (2004). Inequities in Access to Medical Care in Five Countries: Findings from the 2001 Commonwealth Fund International Health Policy Survey. Health Policy.

[21] Hong R., Baumann B.M., Boudreaux E.D. (2007). The Emergency Department for Routine Healthcare: Race/Ethnicity, Socioeconomic Status, and Perceptual Factors. J. Emerg. Med..

[22] Khan Y., Glazier R.H., Moineddin R., Schull M.J. (2011). A Population-Based Study of the Association between Socioeconomic Status and Emergency Department Utilization in Ontario, Canada. Acad. Emerg. Med..

[23] Tozer A.P., Belanger P., Moore K., Caudle J. (2014). Socioeconomic Status of Emergency Department Users in Ontario, 2003 to 2009. CJEM.

[24] Lozano K., Ogbu U.C., Amin A., Chakravarthy B., Anderson C.L., Lotfipour S. (2015). Patient Motivators for Emergency Department Utilization: A Pilot Cross-Sectional Survey of Uninsured Admitted Patients at a University Teaching Hospital. J. Emerg. Med..

[25] Blanchard J., Madden J.M., Ross-Degnan D., Gresenz C.R., Soumerai S.B. (2013). The Relationship between Emergency Department Use and Cost-Related Medication Nonadherence among Medicare Beneficiaries. Ann. Emerg. Med..

[26] Galarraga J.E., Pines J.M. (2016). Costs of ED Episodes of Care in the United States. Am. J. Emerg. Med..

[27] Hunt K.A., Weber E.J., Showstack J.A., Colby D.C., Callaham M.L. (2006). Characteristics of Frequent Users of Emergency Departments. Ann. Emerg. Med..

[28] Diaz R., Walker R.J., Lu K., Weston B.W., Young N., Fumo N., Hilgeman B. (2022). The Relationship between Adverse Childhood Experiences, the Frequency and Acuity of Emergency Department Utilization and Primary Care Engagement. Child Abuse Negl..

[29] Carbone J.T., Jackson D.B., Holzer K.J., Vaughn M.G. (2021). Childhood Adversity, Suicidality, and Non-Suicidal Self-Injury among Children and Adolescents Admitted to Emergency Departments. Ann. Epidemiol..

[30] Bhattarai A., Dimitropoulos G., Marriott B., Paget J., Bulloch A.G.M., Tough S.C., Patten S.B. (2021). Can the Adverse Childhood Experiences (ACEs) Checklist Be Utilized to Predict Emergency Department Visits among Children and Adolescents?. BMC Med. Res. Methodol..

[31] Murray A., Fein J., Beulah B., Mollen C. (2022). Examination of Caregiver Social Factors and Its Influence on Low-Acuity Pediatric Emergency Department Utilization. Pediatr. Emerg. Care.

[32] Cohen-Cline H., Jones K.G., Kulkarni-Rajasekhara S., Polonsky H.M., Vartanian K.B. (2019). Identifying Underlying Constructs of Childhood Adversity in a Low-Income Population. Child Abuse Negl..

[33] Cohen-Cline H., Jones K., Vartanian K. (2021). Direct and Indirect Pathways between Childhood Instability and Adult Homelessness in a Low-Income Population. Child. Youth Serv. Rev..

[34] Gilmer T., Kronick R., Fishman P., Ganiats T. (2001). The Medicaid Rx Model: Pharmacy-Based Risk Adjustment for Public Programs. Med. Care.

[35] Belotti F., Deb P., Manning W.G., Norton E.C. (2015). Twopm: Two-Part Models. Stata J..

[36] Felitti V.J. (2002). The Relation between Adverse Childhood Experiences and Adult Health: Turning Gold into Lead. Perm. J..

[37] Burke N.J., Hellman J.L., Scott B.G., Weems C.F., Carrion V.G. (2011). The Impact of Adverse Childhood Experiences on an Urban Pediatric Population. Child Abuse Negl..

[38] Campbell J.A., Walker R.J., Egede L.E. (2016). Associations between Adverse Childhood Experiences, High-Risk Behaviors, and Morbidity in Adulthood. Am. J. Prev. Med..

[39] Guinn A.S., Ports K.A., Ford D.C., Breiding M., Merrick M.T. (2019). Associations between Adverse Childhood Experiences and Acquired Brain Injury, Including Traumatic Brain Injuries, among Adults: 2014 BRFSS North Carolina. Inj. Prev..

[40] Coster J.E., Turner J.K., Bradbury D., Cantrell A. (2017). Why Do People Choose Emergency and Urgent Care Services? A Rapid Review Utilizing a Systematic Literature Search and Narrative Synthesis. Acad. Emerg. Med..

[41] Xin H. (2019). Patient Dissatisfaction with Primary Care and Nonurgent Emergency Department Use. J. Ambul. Care Manag..

[42] Kangovi S., Barg F.K., Carter T., Long J.A., Shannon R., Grande D. (2013). Understanding Why Patients of Low Socioeconomic Status Prefer Hospitals over Ambulatory Care. Health Aff..

[43] Bieler G., Paroz S., Faouzi M., Trueb L., Vaucher P., Althaus F., Daeppen J.B., Bodenmann P. (2012). Social and Medical Vulnerability Factors of Emergency Department Frequent Users in a Universal Health Insurance System. Acad. Emerg. Med..

[44] Ondler C., Hegde G.G., Carlson J.N. (2014). Resource Utilization and Health Care Charges Associated with the Most Frequent ED Users. Am. J. Emerg. Med..

[45] Moe J., O’Sullivan F., McGregor M.J., Schull M.J., Dong K., Holroyd B.R., Grafstein E., Hohl C.M., Trimble J., McGrail K.M. (2021). Characteristics of Frequent Emergency Department Users in British Columbia, Canada: A Retrospective Analysis. C. Open.

[46] Fruhan S., Bills C.B. (2021). Odds of Return: A Prospective Study Using Provider Assessment to Predict Short-Term Patient Return Visits to the Emergency Department. BMJ Open.

[47] Miller-Cribbs J., Wen F., Coon K., Jelley M., Foulks-Rodriguez K., Stearns J. (2016). Adverse Childhood Experiences and Inequities in Adult Health Care Access. Int. Public Health J..

[48] Hall A., McKenna B., Dearie V., Maguire T., Charleston R., Furness T. (2016). Educating Emergency Department Nurses about Trauma Informed Care for People Presenting with Mental Health Crisis: A Pilot Study. BMC Nurs..

[49] Fischer K.R., Bakes K.M., Corbin T.J., Fein J.A., Harris E.J., James T.L., Melzer-Lange M.D. (2019). Trauma-Informed Care for Violently Injured Patients in the Emergency Department. Ann. Emerg. Med..

[50] Molloy L., Fields L., Trostian B., Kinghorn G. (2020). Trauma-Informed Care for People Presenting to the Emergency Department with Mental Health Issues. Emerg. Nurse.

[51] Chandler G.E., Roberts S.J., Chiodo L. (2015). Resilience Intervention for Young Adults with Adverse Childhood Experiences. J. Am. Psychiatr. Nurses Assoc..

[52] Wang J., Mann F., Lloyd-Evans B., Ma R., Johnson S. (2018). Associations between Loneliness and Perceived Social Support and Outcomes of Mental Health Problems: A Systematic Review. BMC Psychiatry.

[53] Stepanikova I., Oates G.R. (2017). Perceived Discrimination and Privilege in Health Care: The Role of Socioeconomic Status and Race. Am. J. Prev. Med..

[54] Feagin J., Bennefield Z. (2014). Systemic Racism and U.S. Health Care. Soc. Sci. Med..

[55] Richardson L.D., Norris M. (2010). Access to Health and Health Care: How Race and Ethnicity Matter. Mt. Sinai J. Med..

[56] Center for Substance Abuse Treatment (2014). Understanding the Impact of Trauma. Trauma-Informed Care in Behavioral Health Services.

